# Fortified Chestnut Honey Triggers Apoptosis in Colon Cancer Cells

**DOI:** 10.1007/s11130-026-01495-z

**Published:** 2026-04-11

**Authors:** Amaia Iriondo-DeHond, Paloma Morales, Vanesa Sánchez-Martín, Xavier F. Hospital, Manuela Fernández, Eva Hierro, Ana I. Haza

**Affiliations:** 1 Sección Departamental de Nutrición y Ciencia de los Alimentos, Departamento de Nutrición y Ciencia de los Alimentos, Facultad de Veterinaria, Universidad Complutense, Avenida Puerta de Hierro s/n, Madrid, 28040 Spain; 2 Sección Departamental de Farmacia Galénica y Tecnología de los Alimentos, Departamento de Farmacia Galénica y Tecnología de los Alimentos, Facultad de Veterinaria, Avenida Puerta de Hierro s/n, Universidad Complutense, Madrid, 28040 Spain

**Keywords:** Apoptosis pathways, Caco-2 cells, Colon cancer, Chestnut honey, Propolis, Royal jelly

## Abstract

Honey, traditionally a food, is now also used as a complement in cancer therapy due to its anticancer properties. This study aimed to evaluate *in vitro* whether thyme and chestnut honey samples fortified with bee products that had previously shown apoptotic effects on the hepatic system, presented cytotoxic and apoptotic properties in colon cancer (Caco-2) and normal (CCD-18) cells. Cytotoxicity was assessed using the MTT (3-(4,5-dimethylthiazol-2-yl)-2,5-diphenyltetrazolium bromide) assay, while apoptosis was evaluated through cell cycle analysis, annexin V binding, and the activation of DR5 (Death Receptor 5), BAX (BCL-2-Associated X), and caspases 8, 9, and 3. Chestnut honey mixtures selectively triggered apoptosis in colon cancer cells and not in normal cells, being the greatest effect observed in the sample enriched with 10% propolis and 10% royal jelly. Contrary to the effects previously noted in hepatic cells, the apoptotic properties of thyme honey were not improved when fortified with the same bee products, suggesting that the samples exhibit differential anticancer effects depending on the cellular model studied. In this study, selective induction of apoptosis may be attributed to the combined action of phenolic acids and flavones present in this fortified formulation. Samples that induced apoptosis in colon cancer cells did so through both the intrinsic and extrinsic pathways. Fortification of chestnut honey with bee products may represent a promising approach for the development of functional food strategies related to colon cancer.

**Supplementary Information:**

The online version contains supplementary material available at 10.1007/s11130-026-01495-z.

## Introduction

Honey has long been valued for both its nutritional and medicinal properties. It contains a complex matrix of bioactive compounds (including phenolic acids, flavonoids, enzymes, vitamins and minerals) that contributes to its therapeutic potential [1]. The botanical and geographical origin of honey strongly influences its composition, affecting its color, aroma and flavor, and also its bioactive properties [2]. Monofloral honeys, such as thyme and chestnut honey, are renowned not only for their exquisite flavors [3], but also for their health-promoting properties, such as anti-cancer effects [1].

More than half of colon cancer cases are preventable through lifestyle changes, including a healthy diet. Natural products, particularly bee-derived compounds rich in flavonoids and phenolic acids, have shown potential in cancer prevention and therapy. These natural compounds possess different biological activities including antioxidant effects that protect against DNA damage, anti-inflammatory actions that may prevent tumor development, and anti-angiogenic properties that inhibit blood vessel formation necessary for tumor growth [4]. Furthermore, bee products can modulate key cellular pathways regulating cell cycle arrest, apoptosis and metastasis inhibition [5]. The anticancer properties of individual bee products have been widely analyzed; however, very few studies regarding the bioactive properties of bee product mixtures have been published.

Spanish monofloral honeys (chestnut and thyme) enriched with 2–10% propolis and royal jelly have shown greater antioxidant activity than honey samples alone in our previous studies [6]. These antioxidant-rich honey mixtures did not induce apoptosis in normal liver cells, while they enhanced the pro-apoptotic effect of the corresponding honey alone in liver cancer cells [7]. This is of great interest since the strongest anticancer medications often lead to substantial cytotoxicity in healthy tissues [8]. In addition, the differential anticancer activities of natural products are critically important, as their efficacy and mechanism of action often vary significantly depending on the targeted tissue or cancer cell line [9]. Therefore, the present research aimed to evaluate *in vitro* the cytotoxic and apoptotic properties of selected fortified honey samples that had previously shown apoptotic effects on the hepatic system, in colon cancer (Caco-2) and normal (CCD-18) cells.

## Materials and Methods

### Samples

**Fig. 1:**
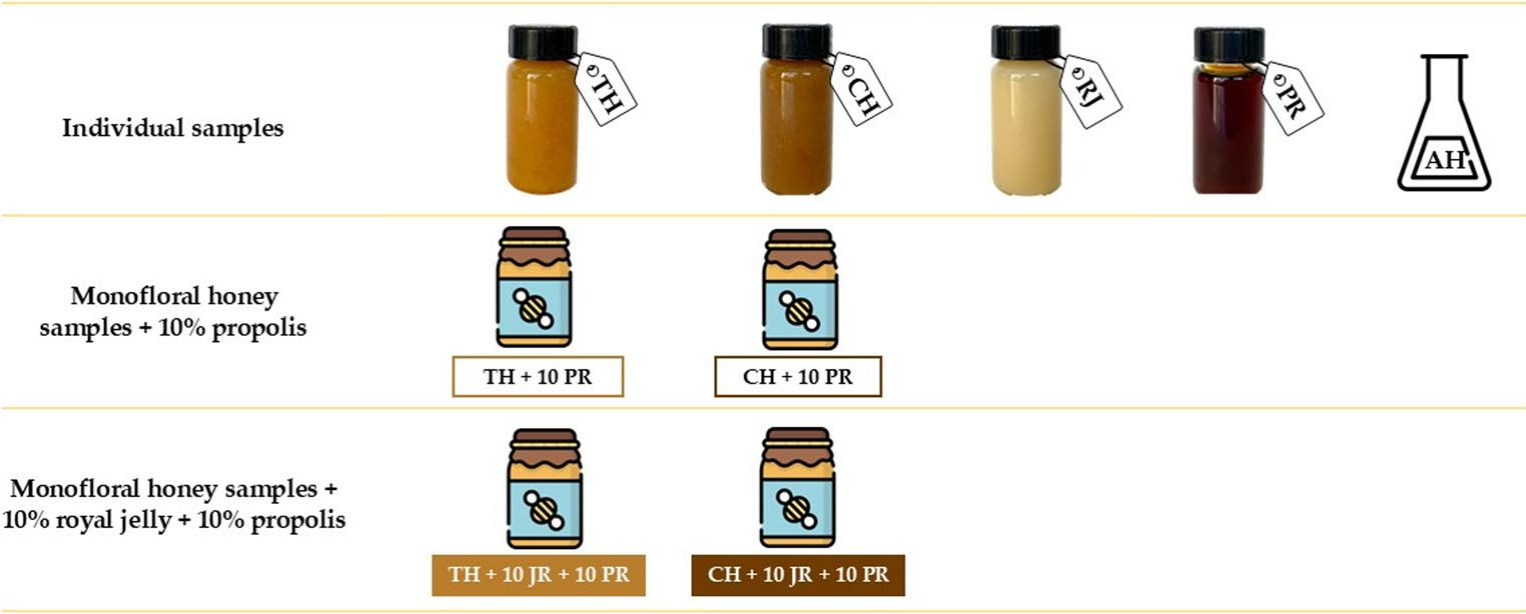
Schematic representation of monofloral honey [thyme (TH) and chestnut (CH)] and bee product samples [royal jelly (RJ) and propolis (PR)] and mixtures (TH+10PR, CH+10PR, TH+10RJ+10PR and CH+10RJ+10PR) analyzed in the present study. Artificial honey (AH) was included as sugar control

This study utilized two monofloral honey samples from Spain as base materials: thyme honey (TH) from Zamora, and chestnut honey (CH) from Toledo. Local beekeepers directly provided these honey samples at the Hive Products Laboratory of the Beekeeping and Agro-Environmental Research Center (CIAPA) in Marchamalo, Guadalajara, Spain. Monofloral honey samples were fortified with royal jelly (RJ) and/or propolis (PR) at 2–10% to obtain 17 samples [6]. Out of the 17 initial samples, only 7 were able to induce apoptosis in liver cancer cells [7], and were selected for this study: TH, CH, TH+10PR, CH+10PR, TH+10RJ+10PR, CH+10RJ+10PR, and PR. Also, RJ and artificial honey (AH) were analyzed (Fig. 1). The physicochemical characterization, total phenolic compounds and antioxidant capacity of these samples have been previously described [6].

### Cell Culture

The Caco-2 cell line, originating from human colorectal adenocarcinoma, was provided by the Instituto de Investigación en Ciencias de la Alimentación (CIAL, CSIC-UAM, Madrid, Spain), and CCD-18 cells, normal human colon tissue cells, were obtained from the Centro de Instrumentación Científica (CIC) of the Universidad de Granada, Spain. Both cell lines were cultured in standard conditions [7].

### MTT Assay

Cell viability was assessed by the MTT assay using the Cell Proliferation Kit I from Roche (Indianapolis, IN, USA). Cells (Caco-2 and CCD-18) were seeded at 1 × 10^5^ and 1 × 10^4^ cells *per* well of a 96-well plate, respectively, and the assay was carried out as described by Sanchez-Martin et al [7].

### Analysis of Apoptosis Induction

Apoptosis induction was evaluated by cell cycle analysis and completed by an annexin-labeled incorporation assay. Caco-2 or CCD-18 cells were cultured at 5 × 10^5^ and 5 × 10^4^ *per* well of a 6-well plate, respectively. Twenty-four hours later, cells were treated with samples (Fig. 1) at 5–100 mg/mL for 24 and 48 h. Negative and positive controls were non-treated cells and cells treated with etoposide 300 µM, respectively. Cell cycle analysis was performed as Sanchez-Martin et al [7]. The percentage of cells in the sub-G1 phase, representing apoptotic or damaged cells, was calculated relative to the total cell number.

On the other hand, induced cell apoptosis was analyzed using the eBioscience™ Annexin V-FITC apoptosis detection kit (Invitrogen, Waltham, MA, USA) following manufacturer’s instructions. Propidium iodide-stained cells were subjected to flow cytometric analysis of apoptosis using a FACS Calibur (Beckton Dickinson, Franklin Lakes, NJ, USA) and Flowing Software 2 (University of Turku, Finland). The percentage of apoptotic cells out of 10⁴ total cells analyzed per experiment was reported and includes populations in the early and late stages of apoptosis.

### Analysis of Apoptotic Pathway Activation

Apoptosis can be activated by the extrinsic (Death Receptor 5, DR5) or the intrinsic (mitochondrial BCL-2-Associated X, BAX) pathway [10]. Caco-2 cells (5 × 10^5^ *per* well) were seeded on a 6-well plate and 24 h later, exposed to TH, CH, CH+10PR, CH+10RJ+10PR and PR at 5–100 mg/mL for 24 and 48 h. Then, DR5 and BAX expression was determined as previously performed by Sánchez-Martín et al [7].

Apoptosis pathway activation was also evaluated by analyzing caspases 8, 9 and 3. To this end, after treatment with samples for 48 h, cell lysates were obtained to analyze caspase activity [7].

### Statistical Analyses

All data are presented as mean ± standard deviation (SD), calculated from three independent experiments. Statistical analysis was performed using one-way analysis of variance (ANOVA), followed by Tukey’s post hoc test for multiple comparisons. Differences were considered statistically significant at *p* < 0.05. All analyses were conducted using Statgraphics Centurion 19 software (Statgraphics Technologies, Inc., The Plains, VA, USA).

## Results and Discussion

### Cytotoxicity of Bee Products and Mixtures on Human Colon Cells

The cytotoxicity of individual bee products (Fig. SM1) and their enriched combinations (Fig. SM2) were evaluated on colon cancer (Caco-2) and normal colon (CCD-18) cell lines. TH resulted cytotoxic at 250 mg/ml in Caco-2 cells (Fig. SM1A) and no cytotoxicity was observed for this sample in CCD-18 cells (Fig. SM1B). Other authors have also reported that other monofloral honey samples, such as Tualang and Gelam honey exerted no cytotoxic effect on normal breast (MCF-10 A) and liver (WRL-68) cells [11]. CH, AH and RJ showed significant cytotoxicity (*p* ≤ 0.001) against both Caco-2 (Fig. SM1 C, E G) and CCD-18 (Fig. SM1 D, F, H) cells only at high concentrations (250 mg/mL) and specifically, RJ was cytotoxic against Caco-2 cells even at 100 mg/ml (Fig. SM1G). CH exhibited slightly higher cytotoxicity than TH, consistent with previous findings in liver cancer cells [12]. It has been reported that the botanical origin highly influences the cytotoxic effect of honey samples on HepG2 and Caco-2 cells [2]. PR showed low cytotoxicity toward Caco-2 cells and no effect on CCD-18 cells (Fig. SM1I, J). Similarly, other studies have found that Thai propolis did not present antiproliferative properties [13].

On the other hand, CH fortified with 10% PR (Fig. SM2C) and with 10% RJ and 10% PR (Fig. SM2G), demonstrated enhanced cytotoxic effects on colon cancer cells compared to TH mixtures (Fig. SM2A and E). Honey and bee product samples and mixtures resulted more cytotoxic to liver than to colon cancer cells [12]. Importantly, none of the bee-product mixtures studied significantly compromised the viability of normal colon cells (Fig. SM2 B, D,F, H). This is of great interest given that most chemotherapeutic agents are toxic to both cancerous and healthy cells, leading to side effects that affect the patient’s life quality [14].

### Evaluation of Apoptosis Induction by Bee Products and Mixtures on Human Colon Cells

**Fig. 2:**
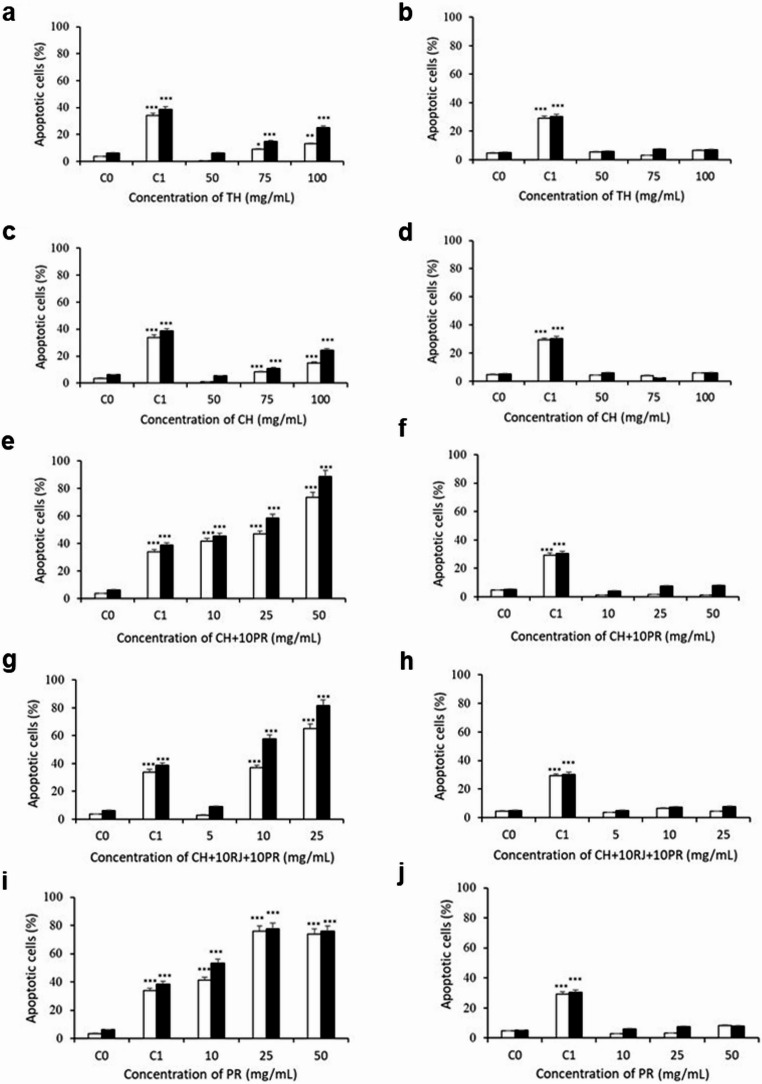
Effect of samples on apoptosis induction of human colon cells analyzed by flow cytometry using the annexin V assay. Caco-2 (**a**, **c**, **e**, **g**, **i**) and CCD-18 (**b**, **d**, **f**, **h**, **j**) cells were treated with different doses (5-100 mg/mL) of TH (**a**, **b**), CH (**c**, **d**), CH+10PR (**e**, **f**), CH+10RJ+10PR (**g**, **h**) and PR (**i**, **j**) for 24 (□) and 48 (■) hours. C_0_, untreated cells; C_1_, cells treated with etoposide (300 µM). Asterisks indicate a significant difference from the control (C_0_). *p ≤ 0.05, **p ≤ 0.01, ***p ≤ 0.001

Figure 2 and Fig. SM3 and SM4 show the apoptotic response of Caco-2 and CCD-18 cells after treatment with TH or CH, respectively. Cell cycle analysis showed a significant increase in Caco-2 cells in the Sub-G1 phase (apoptotic cells) of 13% after treatment with TH or CH (100 mg/mL, 48 h) (Fig. SM3 and SM4A, C). The annexin analysis exhibited that monofloral honey samples induced apoptosis in Caco-2 cells in a concentration- and time-dependent manner (Fig. 2a and c), being the apoptotic cell population of 25% when treated with TH or CH (100 mg/mL, 48 h). No apoptosis induction was observed for normal colon cells (Fig. 2b and d). Despite their different chemical composition, a similar induction of apoptosis (25% induction at 100 mg/mL) was observed when TH and CH were tested on Caco-2 cells. Considering our previous research, TH and CH were more effective in inducing apoptosis in liver cancer cells and almost double the concentration of CH is needed in Caco-2 cells to achieve the same effect in apoptosis induction observed for HepG2 cells. In addition, CH had higher apoptotic properties in liver cancer cells than TH since at 50 mg/mL, CH induced 30% apoptosis and TH did not reach 10% [7]. These findings highlight the differential anticancer effects depending on the cellular model studied, where the same samples cause different responses depending on the cellular context, emphasizing the necessity of evaluating natural products across multiple cancer types [15, 16].

The apoptotic properties of TH were not improved when fortified with bee products (TH+10PR and TH+10JR+10PR) since no significant differences (*p* ≥ 0.01) were observed in Caco-2 cells when compared to control non-treated cells (Table SM1). None of these samples induced apoptosis in normal colon cells. In contrast, our previous results showed that after treatment of HepG2 cells for 48 h, TH+10PR (100 mg/mL) and TH+10JR+10PR (50 mg/mL) induced apoptosis to 30 and 51%, respectively [7]. In agreement, it has been shown that the same bee product can have different apoptotic induction properties depending on the cancer cell type [17]. On the other hand and as observed in liver cancer cells, no apoptosis induction was observed in colon cells for RJ and AH (Table SM1). These results agree with those obtained for liver cells [7], suggesting that apoptosis induction may be related to the phytochemicals and not to the sugars present in honey, such as ferulic, caffeic and benzoic acids, chrysin or luteolin, which possess anticancer properties [18].

The addition of 10% PR or 10% RJ and 10% PR to CH (Fig. 2e and g, respectively) led to a greater apoptotic effect compared to CH alone. After treatment of colon cells with CH+10PR, the number of sub-G1 cells significantly increased in cancer cells at all concentrations for 48 h (12.36% − 54.57%) (Fig. SM5A, C). In the case of CH+10RJ+10PR, an increase up to 20% and 45% was observed in the sub-G1 peak in Caco-2 cells at only 10 and 25 mg/mL for 48 h, respectively (Fig. SM6A, C). The annexin assay confirmed that these samples induced apoptosis in a dose- and time-dependent manner in Caco-2 cells, reaching 90% at the highest time and concentration (50 mg/mL for CH+10PR and 25 mg/mL for CH+10RJ+10PR) (Fig. 2e and g, respectively). CH mixtures did not induce apoptosis in CCD-18 cells, suggesting a selective effect on cancer cells (Fig. 2f and h).

The sub-G1 population in Caco-2 cells increased in a concentration- and time-dependent manner after treatment with PR (26.19%-51.59% at 48 h) (Fig. SM7A, C). Flow cytometry analysis exhibited an increase in apoptotic Caco-2 cells after treatment with PR, exceeding the percentage of apoptotic cells obtained with the positive control etoposide and reaching 77% at 50 mg/mL at 48 h (Fig. 2i). However, this effect was not observed in normal CCD-18 cells (Fig. 2j). Very few studies have been published regarding the effect of bee products on apoptosis induction of normal cell lines, highlighting the need for further research to address this gap. Propolis’ anticancer properties via apoptosis induction have been confirmed *in vitro* in different colon cancer cell lines [4] and *in vivo* [19]. Compounds present in PR such as chrysin, pinocembrin, apigenin, galangin, kaempferol, quercetin, cinnamic acid, o-coumaric acid, p-coumaric acid, caffeic acid and caffeic acid phenylethyl ester (CAPE) have been identified as key contributors to its apoptotic properties [19].

CH combined with bee products induced apoptosis more efficiently than CH, RJ and PR alone. The phenolic profile of some samples used in this study has been previously characterized by our research group [20]. Phenolic compounds from PR such as cinnamic acid derivatives, pinobanksin-3-O-hexanoside, sakuranetin, quercetin-3,7-dimethyl ether and quercetin have been detected in these fortified honey samples [20]. The combination of phenolic acids and flavones present in CH mixtures could be responsible for the large effect of selective apoptosis induction observed in this study. Honey polyphenols are poorly absorbed in the small intestine, and a substantial fraction reaches the colon largely intact, where they may exert direct biological effects on colonocytes and undergo microbial biotransformation into bioavailable catabolites [21–23].

However, the apoptotic effect observed for CH mixtures was not replicated in TH enriched with the same bee products, highlighting the complex and context-dependent nature of polyphenol interactions between honey and bee products [24, 25]. Variable responses are frequently reported for similar food samples, suggesting that the underlying mechanisms governing these polyphenol interactions are still largely undefined [26]. This variability is influenced by multiple factors, including the concentration ratios of specific compounds, the identity of the compounds involved, and the unique physicochemical matrix provided by the honey itself [27].

Our research also indicates that bee product mixtures exhibit differential anticancer effects depending on the cellular model studied. For instance, besides CH, TH and PR alone, enriched TH and CH samples induced apoptosis in liver cancer cells [7], but only CH-enriched mixtures (CH+10PR and CH+10RJ+10PR) had apoptotic effects on colon cancer cells. Following PR, the CH+10RJ+10PR sample exhibited the highest percentage of apoptotic cells in both HepG2 and Caco-2 tumor cell lines [7]. This sample and PR alone showed the highest phenolic content and antioxidant activity and presented protective activity against DNA damage [6, 12]. Chestnut honey mixtures have been employed to obtain nitrate/nitrite-reduced dry fermented sausages with an increased oxidative stability [28]. The incorporation of propolis into honey has been proposed as a strategy to enhance its dietary integration and improve consumer palatability [25]. Also, propolis-fortified honey would enhance the health-promoting properties of honey alone and would reduce costs, as pure propolis is 8 times more expensive than chestnut honey [29].

### Apoptotic Mechanisms Analysis of Samples on Caco-2 Cells

Apoptosis mechanisms of TH and CH, PR and bee product mixtures (CH+10PR and CH+10RJ+10PR) that induced apoptosis in colon cancer cells and not in normal colon cells were then analyzed (Table 1). Only PR was able to induce DR5-positive cells significantly at 24 h, but all samples and etoposide (300 µM), significantly induced (*p* ≤ 0.001) DR5-positive cells at 48 h. High concentrations of TH and CH (100 mg/mL) were needed to reach 32–46% DR5-positive cells. PR only at 10 mg/mL exhibited 95% DR5-positive Caco-2 cells at 48 h. CH+10RJ+10PR activated 78–93% of DR5-positive colon cancer cells at low concentrations and was previously the most effective DR5 activator in liver cancer cells [7]. This effect may be linked to propolis, known to induce apoptosis via TRAIL receptor upregulation, including DR5 [19].

**Table 1:** Effect of bee product samples and etoposide on DR5 and BAX activation in Caco-2 cells, evaluated by flow cytometry at 24 and 48 h

Sample	Concentration (mg/mL)		DR5-positive cells (%)		BAX-positive cells (%)		
			24 h	48 h	24 h		48 h
Control		-	3.44 ± 0.84	4.39 ± 0.82		3.42 ± 0.35	4.92 ± 1.01
TH		75	0.54 ± 0.21	23.72 ± 3.75 ***		6.64 ± 0.78	14.96 ± 0.46 ***
		100	0.62 ± 0.01	32.32 ± 1.05 ***		10.65 ± 2.40 **	14.05 ± 2.33 ***
CH		75	0.42 ± 0.03	32.56 ± 2.12 ***		1.65 ± 1.54	11.08 ± 0.56 ***
		100	1.92 ± 0.30	46.36 ± 2.17 ***		3.25 ± 0.49	12.83 ± 2.36 ***
CH+10PR		10	0.63 ± 0.17	23.71 ± 2.04 ***		1.08 ± 0.16	9.16 ± 2.79 *
		25	1.62 ± 0.42	86.79 ± 2.54 ***		1.21 ± 0.61	13.43 ± 1.01 ***
CH+10RJ+10PR		5	0.89 ± 0.13	78.20 ± 2.61 ***		7.13 ± 1.26	43.44 ± 2.74***
		10	5.60 ± 1.55	93.50 ± 2.19 ***		20.23 ± 1.30 ***	52.9 ± 1.42 ***
PR		10	1.18 ± 0.45	95.36 ± 1.55 ***		2.56 ± 0.75	48.9 ± 1.34 ***
		25	25.60 ± 1.72***	92.12 ± 1.40 ***		3.75 ± 1.07	76.52 ± 2.02 ***
Etoposide		300 µM	1.12 ± 0.28	33.06 ± 2.68 ***		4.08 ± 1.55	16.51 ± 0.68 ***

Data are expressed as the means ± standard deviation (*n* = 3)

Asterisks indicate significant differences from the untreated cells (control)

* *p* ≤ 0.05, ** *p* ≤ 0.01, *** *p* ≤ 0.001

Samples induced BAX-positive cells in a dose- and time-dependent manner (Table 1). TH and CH showed a similar percentage of BAX-positive cells than that obtained for etoposide. Manuka honey also induced apoptosis via BAX protein expression on human colon cancer HCT-116 and LoVo cells [30]. PR was the sample that showed the highest amount of BAX-positive cells, also in HepG2 cells [7]. It has been reported that Iranian propolis increased BAX gene expression in HT-29 cells [31]. Among the two CH mixtures, CH+10RJ+10PR activated five times more BAX-positive cells than CH+10PR. At 5 mg/mL, it increased BAX-positive cells by 43%, similar to the effect of PR at 10 mg/mL.

Results obtained in this study suggest that the tested samples trigger apoptosis in colon cancer cells through both the extrinsic and intrinsic pathways. The higher percentage of DR5-positive cells compared to BAX-positive cells indicates that the extrinsic (death receptor) pathway is mainly activated, while the intrinsic (mitochondrial) pathway may be activated later as a result. This is likely due to the role of Bid, a protein that connects both pathways [17]. When liver and colon cancer cells were treated with these samples, both apoptotic pathways were induced [7].

Caspases 8, 9 and 3 were significantly induced (*p* ≤ 0.001) by all samples (Table 2). Activation of the three caspases analyzed was expected since both apoptotic pathways were activated. Caspase 8 is involved in the extrinsic pathway and caspase 9 in the intrinsic pathway; and both activate the executioner caspase 3. In general, percentage values of caspase 8 were lower in HepG2 than in Caco-2; however, those of caspase 9 were higher [7]. Other research indicated that propolis induced the activation of different caspases in distinct tumor cell lines [32]. Investigating natural anticancer foods that exhibit the capacity to target both the intrinsic and extrinsic apoptotic signaling cascades holds significant promise for efficient cancer therapy. While the results obtained in the present study are promising, they should be interpreted within the limitations of an *in vitro* model, which does not fully replicate the physiological complexity of the human colon.

**Table 2:** Activation of caspases 8, 9, and 3 by bee product samples and etoposide at 48 h

Sample	Concentration (mg/mL)	Caspase 8 (%)	Caspase 9 (%)	Caspase 3 (%)
TH	75	29.37 ± 3.19 ***	33.28 ± 4.04 ***	43.06 ± 3.20 ***
	100	35.81 ± 3.24 ***	42.55 ± 4.60 ***	45.33 ± 2.85 ***
CH	75	38.10 ± 2.99 ***	35.40 ± 3.33 ***	41.91 ± 2.47 ***
	100	38.36 ± 2.98 ***	43.71 ± 3.87 ***	42.21 ± 3.07 ***
CH+10PR	10	41.34 ± 2.70 ***	43.65 ± 2.12 ***	35.02 ± 2.65 ***
	25	44.26 ± 2.42 ***	51.58 ± 2.82 ***	55.39 ± 2.60 ***
CH+10RJ+10PR	5	38.00 ± 2.20 ***	42.84 ± 3.47 ***	40.99 ± 2.48 ***
	10	39.02 ± 2.53 ***	44.72 ± 2.43 ***	45.85 ± 2.42 ***
PR	10	42.92 ± 2.28 ***	32.50 ± 2.99 ***	40.28 ± 2.99 ***
	25	46.89 ± 2.87 ***	38.73 ± 3.96 ***	48.50 ± 2.41 ***
Etoposide	300 µM	43.18 ± 2.47 ***	49.36 ± 3.76 ***	57.99 ± 1.65 ***

Data are expressed as the means ± standard deviation (*n* = 3). The results are expressed as the percentage of caspase activity, assuming the control as 0%

Asterisks indicate significant differences from the untreated cells

***p ≤ 0.001

## Conclusions

In this study, CH, TH, CH mixtures (CH+10PR, CH+10RJ+10PR) and PR selectively induced apoptosis in Caco-2 cells and not in CCD-18 cells. In contrast to the effects previously observed in hepatic cancer cells, apoptotic induction in colon cancer cells was exclusively triggered by chestnut honey mixtures, with the greatest effect observed in the sample fortified with 10% propolis and 10% royal jelly (CH+10RJ+10PR). These findings suggest differential anticancer effects depending on the cellular model studied. The involvement of both the extrinsic and intrinsic pathways in apoptosis induction was confirmed by the activation of DR5 and BAX, and caspases 8, 9 and 3. The combination of phenolic acids and flavones present in CH fortified with 10% propolis and 10% royal jelly may contribute to the large effect of selective apoptosis induction observed in this study. Overall, these *in vitro* results provide a basis for future research into the potential of bee product-enriched chestnut honey in the context of colon cancer prevention and treatment.

## Supplementary Information

Below is the link to the electronic supplementary material.

Supplementary Material 1

## Data Availability

All data supporting the findings of this study are available within the paper and its Supplementary Information.

## Abbreviations

AH: Artificial honey
ANOVA: One-way analysis of variance
BAX: BCL-2-Associated X
CH: Chestnut honey
DR5: Death Receptor 5
MTT: 3-(4,5-dimethylthiazol-2-yl)-2,5-diphenyltetrazolium bromide
PR: Propolis
RJ: Royal jelly
SD: Standard deviation
TH: Thyme honey
