# Supplementary material for: Fortified Chestnut Honey Triggers Apoptosis in Colon Cancer Cells
Source: Plant Foods Hum Nutr. 2026 Apr 11;81(2):45. doi: 10.1007/s11130-026-01495-z (PMC13070085; doi:10.1007/s11130-026-01495-z)
Supplement: Supplementary file 1 — Supplementary Material 1 [file 11130_2026_1495_MOESM1_ESM.docx]

**Fortified chestnut honey triggers apoptosis in colon cancer cells**

Amaia Iriondo-DeHond^1*^, Paloma Morales^1^, Vanesa Sánchez-Martín^1^, Xavier F. Hospital^2^, Manuela Fernández^2^_,_ Eva Hierro^2^, Ana I. Haza^1^ ***Corresponding author:** Amaia Iriondo-DeHond, [amaiairi@ucm.es](mailto:amaiairi@ucm.es)

^1^ Sección Departamental de Nutrición y Ciencia de los Alimentos, Departamento de Nutrición y Ciencia de los Alimentos, Facultad de Veterinaria, Universidad Complutense, Avenida Puerta de Hierro s/n, 28040 Madrid, Spain.

^2^ Sección Departamental de Farmacia Galénica y Tecnología de los Alimentos, Departamento de Farmacia Galénica y Tecnología de los Alimentos, Facultad de Veterinaria, Avenida Puerta de Hierro s/n, Universidad Complutense, 28040 Madrid, Spain.

**Supplementary Results**

**
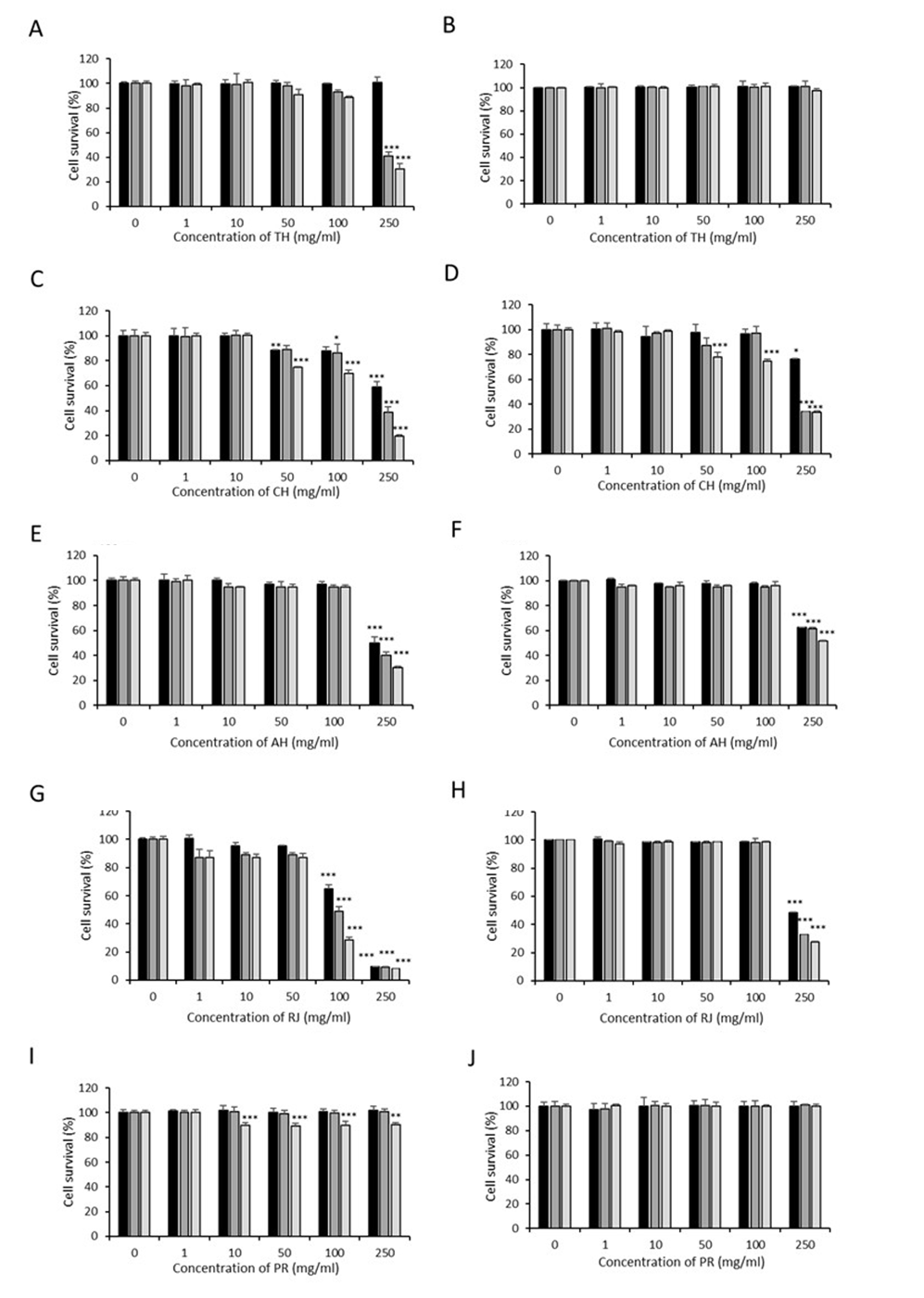
**

**Fig. SM1** Effect on the viability of Caco-2 (A, C, E, G, I) and CCD-18 (B, D, F, H, J) cells of TH (A, B), CH (C, D), AH (E, F), RJ (G, H), and PR (I, J). Cells were cultured with different doses of samples (0-250 mg/ml) for 24 (■), 48 (■), and 72 hours (■). Asterisks indicate a significant difference from the control (0). * *p* ≤ 0.05, ** *p* ≤ 0.01, *** *p* ≤ 0.001.

**
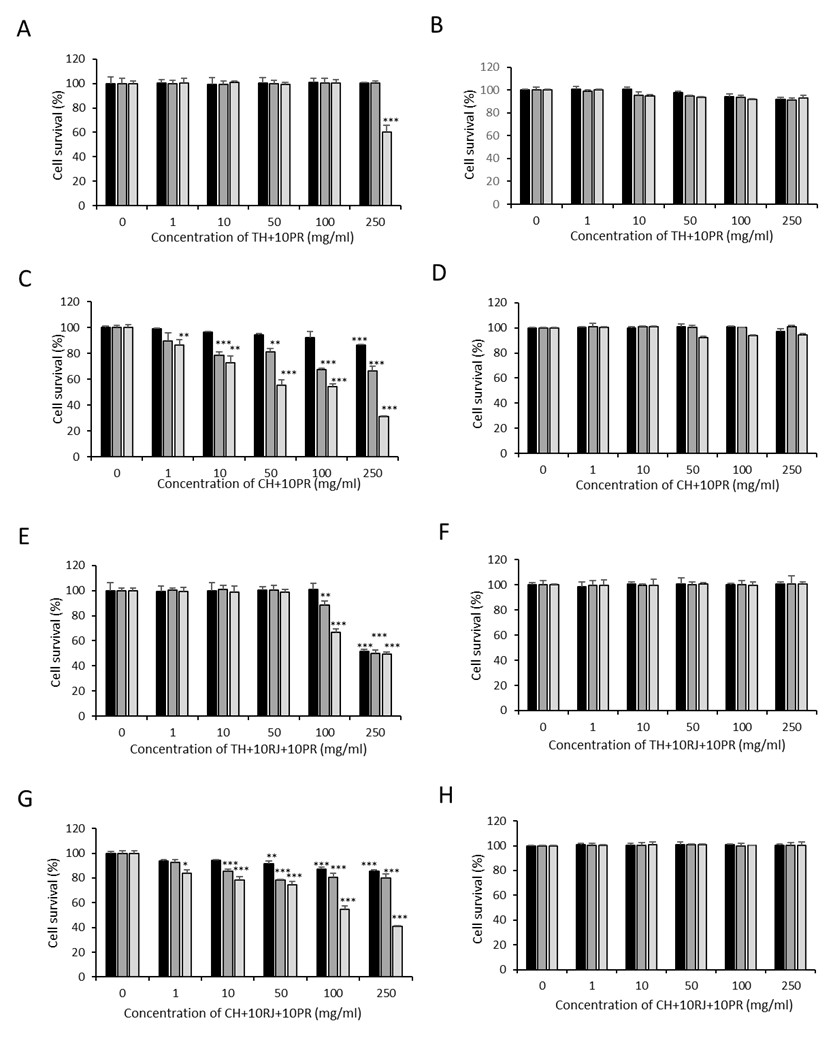
**

**Fig. SM2** Effect on the viability of Caco-2 (A, C, E, G) and CCD-18 (B, D, F, H) cells of TH+10PR (A, B), CH+10PR (C, D), TH+10RJ+10PR (E, F) and CH+10RJ+10PR (G, H). Cells were cultured with different doses of samples (0-250 mg/ml) for 24 (■), 48 (■), and 72 hours (■). Asterisks indicate a significant difference from the control (0). * *p* ≤ 0.05, ** *p* ≤ 0.01, *** *p* ≤ 0.001.

**
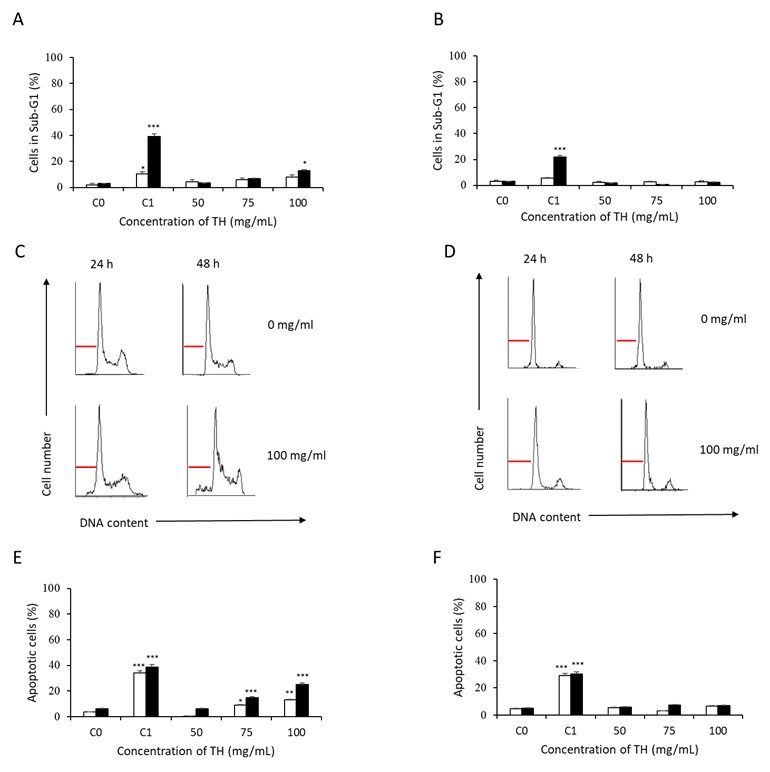
**

**Fig. SM3** Effect of TH on the cell cycle of human colon cells determined by flow cytometry. Caco-2 (A) and CCD-18 (B) cells were treated with different doses of TH (50, 75, and 100 mg/mL) for 24 (□) and 48 (■). Representative histograms of the cell cycle of Caco-2 (C) and CCD-18 (D) cells are shown; the red line indicates the Sub-G1 phase. C_0_, untreated cells; C_1_, cells treated with etoposide (300 µM). Asterisks indicate a significant difference from the control (C_0_). **p≤0.05, **p≤0.01, ***p≤0.001*.


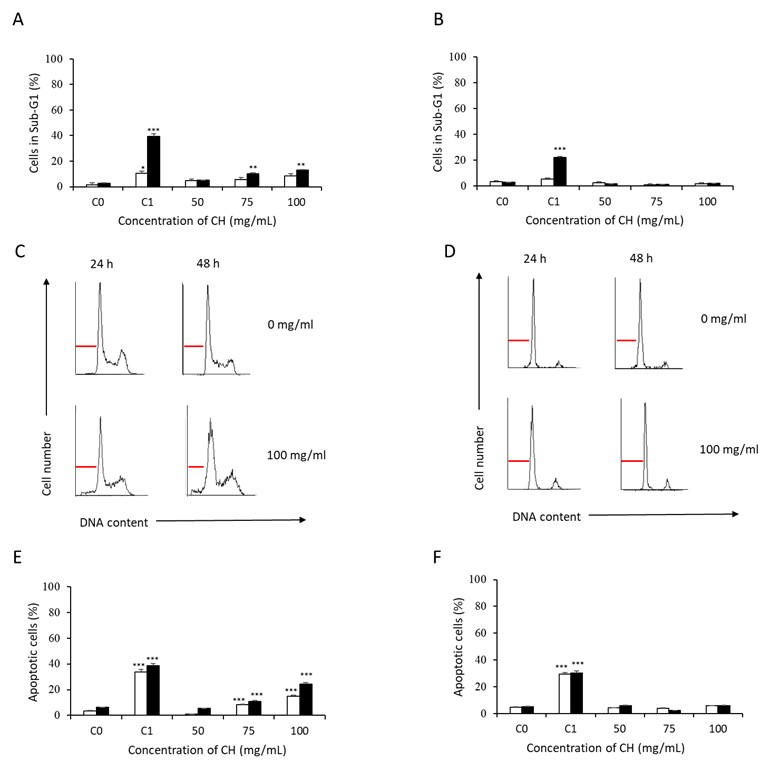


**Fig. SM4** Effect of CH on the cell cycle of human colon cells determined by flow cytometry. Caco-2 (A) and CCD-18 (B) cells were treated with different doses of CH (50, 75, and 100 mg/mL) for 24 (□) and 48 (■). Representative histograms of the cell cycle of Caco-2 (C) and CCD-18 (D) cells are shown; the red line indicates the Sub-G1 phase. C_0_, untreated cells; C_1_, cells treated with etoposide (300 µM). Asterisks indicate a significant difference from the control (C_0_). **p≤0.05, **p≤0.01, ***p≤0.001*.

**Table SM1** Effect of TH+10PR, TH+10RJ+10PR, RJ and AH (50, 75 and 100 mg/mL at 24 and 48 h) on human colon cells apoptosis analyzed by flow cytometry using the annexin V assay.

| **Sample** | **Concentration**  **(mg/mL)** | **Apoptotic cells (%)**  **Caco-2** | | **Apoptotic cells (%)**  **CCD-18** | |
| --- | --- | --- | --- | --- | --- |
|  |  | **24 h** | **48 h** | **24 h** | **48 h** |
| Control | - | 3.55 ± 0.70 | 6.14 ± 1.34 | 3.68 ± 0.14 | 5.11 ± 0.69 |
| Etoposide | 300 µM | 33.95 ± 1.05 *** | 38.57 ± 1.18 *** | 29.26 ± 1.48 ** | 30.31 ± 1.95 ** |
| TH+10PR | 50 | 3.34 ± 0.56 | 8.87 ± 0.18 | 3.53 ± 0.48 | 5.93 ± 0.61 |
|  | 75 | 4.62 ± 0.74 | 9.92 ± 0.78 | 5.09 ± 0.98 | 6.01 ± 0.84 |
|  | 100 | 6.33 ± 0.28 | 9.96 ± 0.63 | 5.93 ± 0.87 | 8.41 ± 2.68 |
| TH+10RJ+10PR | 50 | 1.83 ± 0.99 | 5.46 ± 1.16 | 4.24 ± 0.23 | 6.35 ± 0.28 |
|  | 75 | 2.98 ± 0.64 | 8.68 ± 0.15 | 5.42 ± 0.27 | 8.01 ± 0.78 |
|  | 100 | 5.55 ± 0.39 | 9.70 ± 1.18 | 5.45 ± 0.49 | 8.22 ± 0.61 |
| RJ | 50 | 1.19 ± 0.99 | 1.30 ± 0.56 | 3.02 ± 0.12 | 2.35 ± 0.12 |
|  | 75 | 1.30 ± 0.44 | 1.60 ± 0.11 | 5.09 ± 2.18 | 5.22 ± 0.24 |
|  | 100 | 1.40 ± 0.35 | 1.50 ± 0.91 | 5.12 ± 0.24 | 5.30 ± 0.18 |
| AH | 50 | 1.17 ± 0.21 | 1.22 ± 0.32 | 3.63 ± 0.87 | 4.10 ± 0.72 |
|  | 75 | 1.10 ± 0.64 | 1.15 ± 0.24 | 4.34 ± 0.13 | 5.11 ± 0.37 |
|  | 100 | 1.11 ± 0.32 | 1.49 ± 0.55 | 5.30 ± 0.84 | 6.03 ± 0.74 |

Data are expressed as the means ± standard deviation (n = 3). Asterisks indicate significant differences from the untreated cells. ***p≤0.01, *****p≤0.001*.

**
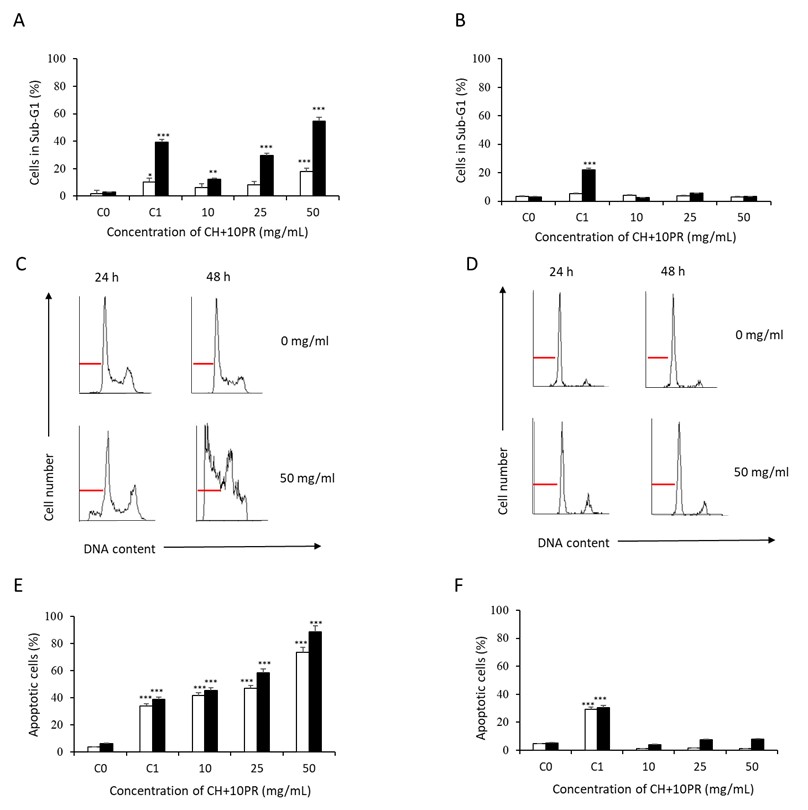
**

**Fig. SM5** Effect of CH+10PR on the cell cycle of human colon cells determined by flow cytometry. Caco-2 (A) and CCD-18 (B) cells were treated with different doses of CH+10PR (10, 25 and 50 mg/mL) for 24 (□) and 48 (■). Representative histograms of the cell cycle of Caco-2 (C) and CCD-18 (D) cells are shown; the red line indicates the Sub-G1 phase. C_0_, untreated cells; C_1_, cells treated with etoposide (300 µM). Asterisks indicate a significant difference from the control (C_0_). **p≤0.05, **p≤0.01, ***p≤0.001*.


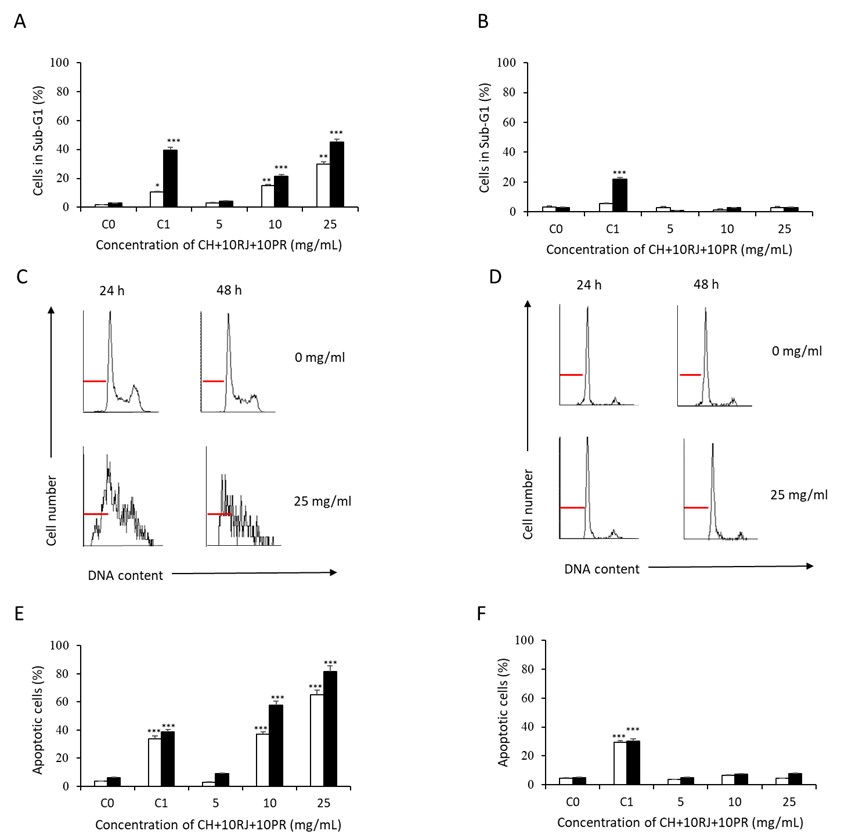


**Fig. SM6** Effect of CH+10RJ+10PR on the cell cycle of human colon cells determined by flow cytometry. Caco-2 (A) and CCD-18 (B) cells were treated with different doses of CH+10RJ+10PR (5, 10, and 25 mg/mL) for 24 (□) and 48 (■). Representative histograms of the cell cycle of Caco-2 (C) and CCD-18 (D) cells are shown; the red line indicates the Sub-G1 phase. C_0_, untreated cells; C_1_, cells treated with etoposide (300 µM). Asterisks indicate a significant difference from the control (C_0_). **p≤0.05, **p≤0.01, ***p≤0.001*.

**
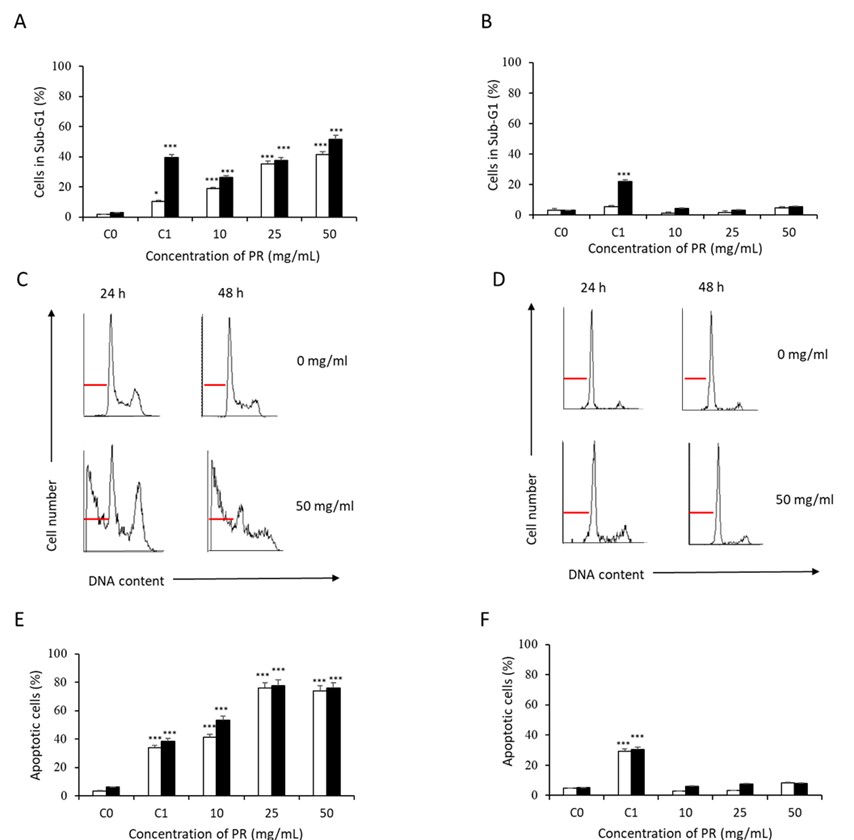
**

**Fig. SM7** Effect of PR on the cell cycle of human colon cells determined by flow cytometry. Caco-2 (A) and CCD-18 (B) cells were treated with different doses of PR (10, 25, and 50 mg/mL) for 24 (□) and 48 (■). Representative histograms of the cell cycle of Caco-2 (C) and CCD-18 (D) cells are shown; the red line indicates the Sub-G1 phase. C_0_, untreated cells; C_1_, cells treated with etoposide (300 µM). Asterisks indicate a significant difference from the control (C_0_). **p≤0.05, **p≤0.01, ***p≤0.001.*
